# Scribner’s Monthly, an Illustrated Magazine for the People

**Published:** 1873-02

**Authors:** 


Scribner's Monthly, an Illustrated Magazine for the People.—The February number of this magazine is a gem. The illustrations are beautiful and contents varied, interesting and instructive. This number alone is worth the price of subscription. Scribner's is edited by Dr. J. G. Holland—the poet, scholar and divine—whose story, "Arthur Bonnicastle," is now its most attractive feature. A more charming and interesting story we have seldom read. Those of our readers desiring a first-class magazine can find it in Scribner's. Price, four dollars. Address, Scribner & Co., 654 Broadway, New York.

